# Clinical Effectiveness of Oral Relugolix in Advanced Prostate Cancer: A Structured Review of Current Primary Research

**DOI:** 10.7759/cureus.97231

**Published:** 2025-11-19

**Authors:** Adnan Higgi, Carys Melvin, Ahmed Abdelrasheed, Katherine Wilson

**Affiliations:** 1 Urology, Royal Glamorgan Hospital, Llantrisant, GBR

**Keywords:** androgen deprivation therapy, drug efficacy, prostate cancer, relugolix, treatment effect

## Abstract

Relugolix is a novel orally administered gonadotropin-releasing hormone (GnRH) antagonist approved for androgen deprivation therapy (ADT) in advanced prostate cancer. Its oral formulation, rapid onset of action, and potentially improved cardiovascular safety profile distinguish it from traditional injectable GnRH antagonists. This review evaluates current primary research on the clinical effectiveness and safety of Relugolix compared to established ADT agents. A structured literature search was conducted using PubMed, targeting primary research articles that assessed the efficacy and safety of oral Relugolix. Inclusion criteria comprised original studies with clinical endpoints such as testosterone suppression, prostate-specific antigen (PSA) response, and castration resistance-free survival (CRFS). Exclusion criteria included reviews, meta-analyses, and studies not investigating Relugolix. Searches were completed by two independent reviewers compared following the Preferred Reporting Items for Systematic Reviews and Meta-Analyses guidelines. Nine primary studies met the inclusion criteria, including randomized controlled trials (RCTs), subgroup analyses, pharmacokinetic modelling, and observational studies. In the HERO trial, Relugolix achieved castration-level testosterone (<50 ng/dL) in 96.7% of patients, outperforming Leuprolide (88.3%) in both speed (median = 4 days vs. 29 days) and magnitude of suppression. Subgroup analyses demonstrated consistent efficacy across patients receiving concomitant therapies with a lower incidence of major cardiovascular events in the Relugolix group. Additional studies confirmed its effectiveness when combined with radiotherapy in comparison with Degarelix. Pharmacokinetic modelling supported rapid and sustained testosterone suppression even during short treatment interruptions. Relugolix is an effective and well-tolerated oral GnRH antagonist for patients with advanced prostate cancer. It offers rapid testosterone suppression, high rates of CRFS, and a potentially favorable cardiovascular safety profile compared to injectable ADT agents. These advantages, along with oral administration, support its use as a viable alternative in clinical practice. Further long-term studies are warranted to confirm sustained outcomes and optimize treatment regimes.

## Introduction and background

Prostate cancer remains one of the most prevalent malignancies affecting men worldwide. It represents the second most common solid malignancy in the male population [1]. The disease can be categorized into localized disease, locally advanced, non-metastatic castration-resistant prostate cancer, and metastatic disease [2]. Advanced stages of the disease often require systemic therapy to limit the progression and spread of the malignancy. These include chemotherapy agents, radiotherapy, and androgen deprivation therapy (ADT) [3]. ADT has been shown to be effective in the treatment of both metastatic and non-metastatic disease. ADTs exert their effect on cancers by suppressing circulating testosterone and subsequently suppressing tumor growth [4].

Traditionally, ADTs have been administered via injectable gonadotropin-releasing hormone (GnRH) antagonists, such as Leuprolide and Degarelix. However, these formulations have a delayed onset of action and a significant cardiovascular side-effect profile [5]. Relugolix is the first licensed oral GnRH receptor antagonist approved for the treatment of prostate cancer in 2020 [6]. Relugolix acts as a GnRH receptor antagonist, suppressing testosterone production by inhibiting luteinizing hormone and follicle-stimulating hormone release [7].

The approval of Relugolix was largely driven by the pivotal HERO trial, which demonstrated its advantage over Leuprolide in key clinical outcomes [7]. Since then, additional studies have aimed to assess the efficacy and side-effect profile compared to alternative GnRH antagonists.

This review assesses current evidence from primary research in the literature to evaluate the clinical effectiveness of Relugolix by examining its performance relative to other established ADT agents. We aim to provide clinicians with a comprehensive understanding of the current research into Relugolix to help guide decision-making in the treatment of advanced prostate cancer. As a secondary objective, we reviewed the safety data from the included trials to help increase awareness of its tolerability profile and inform risk-benefit discussions in clinical practice. This review has not previously been published or presented at any major conferences, either in abstract or full.

## Review

Methodology

This review followed the Preferred Reporting Items for Systematic Reviews and Meta-Analyses (PRISMA) 2020 guidelines. A structured literature review was conducted via PubMed and Cochrane Library to identify relevant articles assessing the efficacy of oral Relugolix. The search strategy was developed using the PubMed advanced search builder and combined three thematic lines with Boolean operators (Table 1). The review process adhered to PRISMA 2020 guidelines by clearly reporting the eligibility criteria, information sources, and study selection process. Records were screened by title and abstract, followed by full-text assessment for inclusion. The study selection process was documented using a PRISMA 2020-compliant flow diagram. Data extraction and analysis were performed in accordance with PRISMA recommendations, with key findings summarized systematically. We aimed to evaluate data exclusively from primary research articles.

**Table 1:** Search terms used to develop the Boolean operators.

Line	Search terms
#1	Prostatic neoplasms[majr] OR cancer, prostate[majr] OR ((prostate[tiab] OR prostatic[tiab] AND cancer[tiab])
#2	Relugolix[All Fields] OR Orgovyx[tiab] OR Myfembree[tiab] OR Ryeqo[tiab]
#3	efficacy[tiab] OR effectiveness[tiab] OR treatment efficiacy[tiab] OR clinical effectiveness[tiab] OR therapeutic outcome[tiab] OR treatment response[tiab] OR clinical response[tiab] OR disease progression[tiab] OR overall survival[tiab] OR mortality[Mesh] OR mortality[tiab]
#1+2+3	((Prostatic neoplasms[majr] OR cancer, prostate[majr] OR ((prostate[tiab] OR prostatic[tiab] AND cancer[tiab])) AND (“relugolix”[All Fields] OR Orgovyx[tiab] OR Myfembree[tiab] OR Ryeqo[tiab])) AND (efficacy[tiab] OR effectiveness[tiab] OR treatment efficiacy[tiab] OR clinical effectiveness[tiab] OR therapeutic outcome[tiab] OR treatment response[tiab] OR clinical response[tiab] OR disease progression[tiab] OR overall survival[tiab] OR mortality[Mesh] OR mortality[tiab]))

Inclusion Criteria

Only primary research articles were included. Eligible study designs included randomized controlled trials (RCTs), prospective or retrospective cohort studies, case-control studies, and preclinical experimental studies. Studies were required to investigate Relugolix as the main therapeutic intervention. Trials assessing Relugolix either as a monotherapy or in combination with other agents were included. Articles needed to report outcomes related to the efficacy, such as testosterone suppression, disease progression, or PSA response. Alternatively, articles needed to evaluate the safety profile of Relugolix, such as adverse events, tolerability, or cardiovascular risk. Studies involving human participants with prostate cancer were included. Preclinical studies using animal models or cell lines relevant to prostate cancer were also eligible if they assessed the pharmacological activity or toxicity of Relugolix. Only articles published in peer-reviewed journals and available in English were included.

Exclusion Criteria

Articles that were not primary research, including literature reviews, systematic reviews, meta-analyses, editorials, commentaries, and conference abstracts without full data, were excluded. Studies that did not evaluate Relugolix or only mentioned it incidentally without investigating its effects were excluded. Research focusing exclusively on other GnRH antagonists or agonists (e.g., Leuprolide, Degarelix) without a Relugolix arm was excluded. Articles that did not report relevant efficacy or safety outcomes were excluded. Duplicate publications or secondary analyses of previously reported data were excluded unless they provided new or distinct outcomes.

Data Extraction

Two independent reviewers performed a comprehensive literature search and data extraction process in accordance with the predefined inclusion and exclusion criteria. Searches were performed using advanced search functions within the electronic databases PubMed and the Cochrane Library.

**Figure 1:**
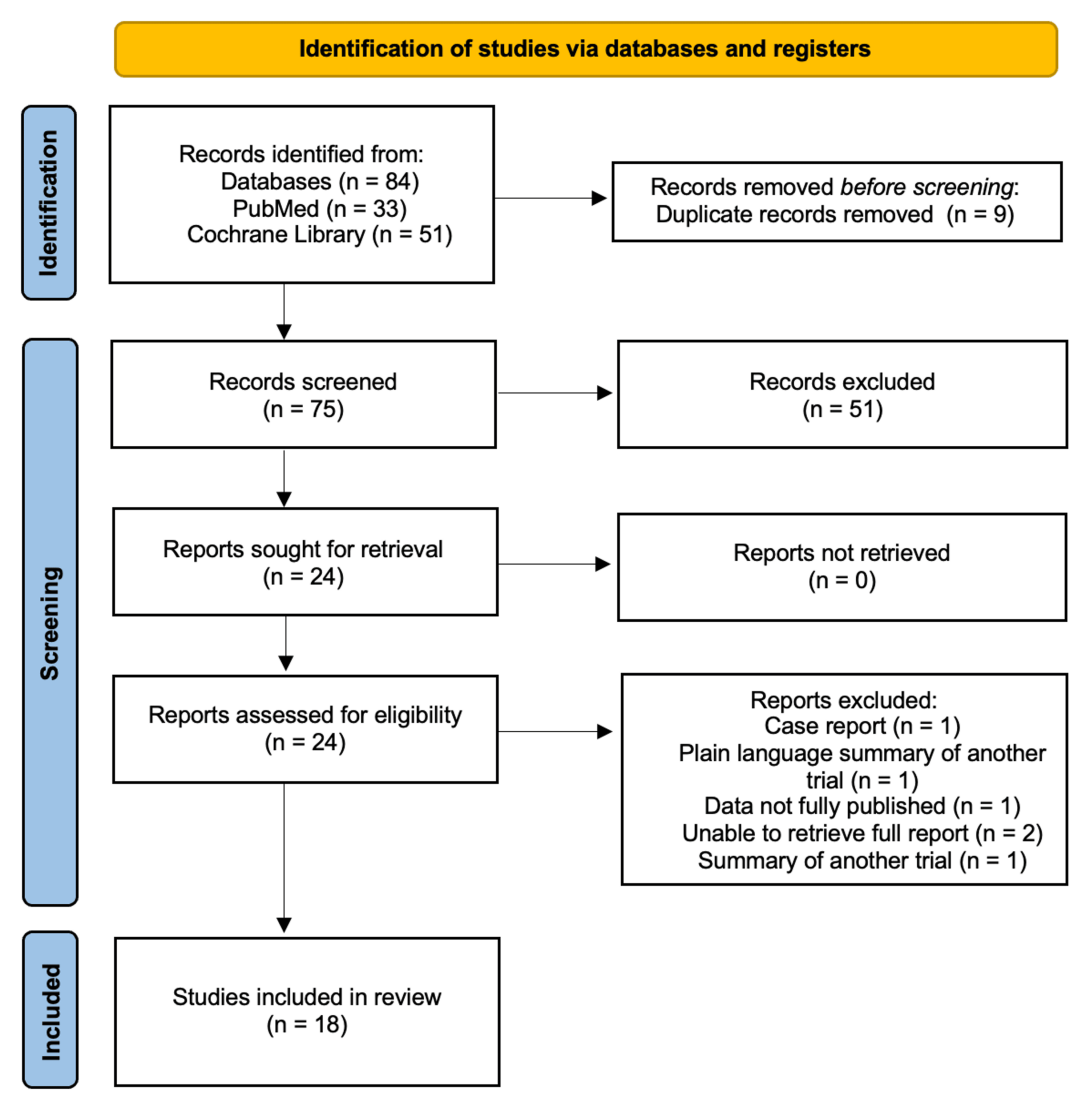
Preferred Reporting Items for Systematic Reviews and Meta-Analyses (PRISMA) flowchart demonstrating the search methodology.

The initial search identified 84 articles. All retrieved records were imported into a reference management tool (Zotero) for organization, and duplicate records were removed. A total of nine duplicate records were identified and excluded, leaving 75 studies for screening. Titles and abstracts of these studies were then independently reviewed by both reviewers to assess eligibility based on the established criteria. Following this initial screening, 51 studies were excluded for not meeting the inclusion criteria. The remaining 24 articles underwent a full-text review to determine their suitability for inclusion by each reviewer. Discrepancies between reviewers were resolved through discussion. After full-text assessment, six additional studies were excluded. The specific reasons are documented in the PRISMA flow diagram (Figure 1). In total, 18 studies were included for analysis in this review. The data collected from each study included study characteristics, population details, comparators, outcomes, and follow-up duration.

Results

In total, 18 primary research articles were selected for final analysis and review. Table 2 illustrates each study in more detail, including population size, study design, and study duration.

**Table 2:** Summary of all articles included in the review.

Authors	Year	Country	Sample size	Study duration	Study type
Shore et al. [6]	2020	Multinational	930	48 weeks	Subgroup analysis of a phase 3 trial
Saad et al. [7]	2023	Multinational	1,508	48 weeks	Phase 3 randomized controlled trial
George et al. [8]	2023	Multinational	934	48 weeks	Subgroup analysis of a phase 3 trial
Spratt et al. [9]	2024	USA, France, Belgium	260	24–48 weeks	Post hoc analysis
Lee et al. [10]	2023	USA, Switzerland	Multiple trials	Combined analysis	Modelling study
Dearnaley et al. [11]	2020	USA	103	12 months	Randomized controlled trial
Gallagher et al. [12]	2023	USA	52	6.2 months	Prospective observational study
Shore et al. [13]	2023	USA	930	48 weeks	Retrospective observational study
Xia et al. [14]	2024	Multinational	5,059 reports	January 2021–March 2023	Post-marketing disproportionality analysis
Brown et al. [15]	2023	USA	12	28 days + follow-up	Phase II open-label substudy
Bossi et al. [16]	2021	Multinational	930	48 weeks	Subgroup analysis of a phase 3 trial
Cookson et al. [17]	2021	Multinational	930	48 weeks	Subgroup analysis of a phase 3 trial
George et al. [18]	2022	Multinational	930	48 weeks	Subgroup analysis of a phase 3 trial
Saad et al. [19]	2022	Multinational	930	48 weeks	Subgroup analysis of a phase 3 trial
Ye et al. [20]	2022	Asia	297	48 weeks	Subgroup analysis of a phase 3 trial
Tombal et al. [21]	2023	Multinational	930	48 weeks	Post ad hoc analysis
Bailen et al. [22]	2022	Multinational	1,012	48 weeks	Pooled safety analysis
Patel et al. [23]	2024	Multinational	65	24 months	Randomized control trial

Clinical Efficacy

In the HERO trial, Shore et al. performed a 2:1 RCT comparing Relugolix (n = 622) to Leuprolide (n = 308) in a population of 930 men. Relugolix achieved castration-level testosterone (<50 ng/dL) in 96.7% (95% confidence interval (CI) = 94.9 to 97.9) compared to 88.3% (95% CI = 84.6 to 91.8) with Leuprolide. When evaluating secondary endpoints, the study demonstrated overall superiority of the Relugolix group compared to the Leuprolide group (p < 0.001). The cumulative probability of achieving castration by day four was 56.0% in the Relugolix group versus 0% in the Leuprolide group, and by day 15, it was 98.7% versus 12.0%, respectively. Additionally, testosterone suppression to profound castrate levels (<20 ng/dL) by day 15 was achieved in 78.4% of patients receiving Relugolix compared to 1.0% receiving Leuprolide. Additionally, PSA response rates were higher for those receiving Relugolix (79.4% vs. 19.8%; p < 0.001) [6].

Building on the HERO trial, Saad et al. analyzed a total of 1,508 participants prescribed either Relugolix or Leuprolide for prostate cancer. Overall, 1,074 were diagnosed with advanced prostate cancer and 434 with metastatic prostate cancer. The study assessed a primary endpoint of castration-level testosterone (<50 ng/dL) and a secondary endpoint of castration resistance-free survival (CRFS) during 48 weeks. The sustained castration rate at 48 weeks, comparing the Relugolix and Leuprolide groups, was 91.7% and 97.0%, respectively (95% CI = −13.7%, 3.0%). The study also demonstrated that overall CRFS rates were 86.8% (95% CI = 84.0%, 89.2%) in the Relugolix group and 87.3% (95% CI = 83.2%, 90.5%) in the Leuprolide group (hazard ratio (HR) = 1.03 (0.72, 1.49); p = 0.89) [7].

Subgroup Analysis

George et al. aimed to assess the efficacy and safety of concomitant medication alongside Relugolix in patients with advanced prostate cancer. The analysis found that efficacy was maintained across all subgroups when compared to the population in the HERO trial. This included concomitant prostate cancer medications such as docetaxel and enzalutamide. In patients receiving enzalutamide or docetaxel alongside ADT, sustained castration rates (<50 ng/dL) from day 29 to week 48 were 95.8% with Relugolix and 90.9% with leuprolide. Among those not receiving these additional treatments, castration rates were 96.7% for Relugolix and 88.7% for Leuprolide. These results demonstrated that Relugolix consistently achieved higher castration rates than Leuprolide, regardless of whether patients received additional prostate cancer treatments such as enzalutamide or docetaxel [8].

Radiotherapy and Adjuvant/Neoadjuvant Treatment

A further study by Spratt et al. assessed the efficacy of combining GnRH antagonist with radiotherapy on CRFS. No statistically significant difference was demonstrated between the Relugolix and Leuprolide groups (HR = 0.97; 95% CI = 0.35-2.72; p = 0.62). The study was split into those receiving short-term ADT therapy (24 weeks) and those receiving longer-term ADT therapy (48 weeks). Relugolix achieved castration rates of 95% (95% CI = 87.1%-99.0%) and 97% (95% CI = 90.6%-99.0%) among patients receiving short-term and longer-term ADT, respectively. Relugolix combined with radiotherapy showed a favorable PSA response and testosterone suppression [9].

Modelling Study

A population pharmacokinetic/pharmacodynamic model developed by Lee et al. confirmed rapid onset and sustained suppression of testosterone with Relugolix. Simulations confirmed that Relugolix achieves castration-level testosterone (<50 ng/dL) by day two and profound castration (<20 ng/dL) by day nine of treatment. Additionally, the model demonstrated maintenance of testosterone suppression even during temporary treatment interruptions. After a seven-day break, 97.3% of patients maintained suppression, and 85.5% maintained suppression after a 14-day break [10].

Clinical Efficacy of Relugolix Versus Degarelix

Dearnaley et al. conducted an RCT including 103 intermediate-risk patients undergoing primary external beam radiation therapy and neoadjuvant/adjuvant ADT. The study found that Relugolix achieved higher castration rates than Degarelix at both testosterone thresholds (95% vs. 89% at 1.73 nmol/L and 82% vs. 68% at 0.7 nmol/L). Median time to castration with Relugolix was four days. Both treatments reduced PSA levels and prostate volume. Three months post-treatment, testosterone recovery occurred in 52% of Relugolix patients versus 16% with Degarelix. Quality of life scores improved after discontinuation. Hot flushes were the most common adverse event (Relugolix = 57%, Degarelix = 61%) [11].

Neoadjuvant and Adjuvant Relugolix Alongside Stereotactic Body Radiation Therapy

A prospective study by Gallagher et al. evaluated early biochemical outcomes in 52 men with intermediate or high-risk prostate cancer. The patients were treated with neoadjuvant/adjuvant Relugolix alongside stereotactic body radiation therapy (SBRT). The combination demonstrated high rates of testosterone suppression, with both effective (≤50 ng/dL) and profound (≤20 ng/dL) castration rates at the time of SBRT and in early follow-up. PSA levels also declined significantly across all risk groups, with 87.2% of patients achieving a PSA of ≤0.5 ng/mL and 74.4% reaching ≤0.2 ng/mL within four months after SBRT [12].

Safety Profile of Relugolix in Prostate Cancer Management

Across multiple clinical studies, Relugolix has demonstrated a favorable safety profile consistent with other forms of ADT. The most frequently reported adverse event was hot flushes, occurring in approximately 50-60% of patients, followed by gastrointestinal symptoms such as diarrhea. This was more common with Relugolix than with Leuprolide but was generally mild and non-disruptive [6-8].

Importantly, in the Phase 3 HERO trial, major adverse cardiovascular events (MACE) were significantly lower in the Relugolix group compared to Leuprolide (2.9% vs. 6.2%) (HR = 0.46; 95% CI = 0.24 to 0.88) [6]. This suggests a potential cardiovascular benefit, particularly in patients with pre-existing cardiovascular conditions. Fatal adverse events were also less frequent with Relugolix versus Leuprolide (1.1% vs. 2.9%) [13]. When combined with radiotherapy, Relugolix maintained a consistent safety profile. The most common adverse events remained hot flushes, fatigue, and gastrointestinal complaints. Severe adverse events were infrequent (<5%) and included isolated cases of hypertension and headache [11].

A post-marketing safety analysis of Relugolix using the FDA Adverse Event Reporting System by Xia et al. demonstrated the most frequently reported adverse events. These included hot flushes, fatigue, headache, asthenia, dizziness, hypertension, diarrhea, palpitations, depression, and insomnia [14]. Among these, hot flushes and fatigue were the most commonly reported adverse events. This is consistent with findings from other clinical trials [6,9,13]. Cardiovascular-related events such as hypertension and palpitations were reported but did not show a disproportional increase, supporting the favorable cardiovascular safety profile observed in the HERO trial [6,13]. Serious adverse events were rare, and fatal outcomes were infrequent. Xia et al. reported several other adverse events, such as erectile dysfunction and gynecomastia [14].

Open-Label Phase II Trial

In a 2023 study by Brown et al., the co-administration of Relugolix and Apalutamide was evaluated in 12 men with high-risk localized prostate cancer following radical prostatectomy. Patients initially received Relugolix monotherapy (360 mg loading dose followed by 120 mg daily for 14 days) before adding apalutamide (240 mg daily) for 28 days. After two weeks of Relugolix alone, all participants achieved castrate testosterone levels (<50 ng/dL), with median testosterone dropping from 348.5 ng/dL at baseline to 8.7 ng/dL. During combination therapy, 11 of 11 patients maintained castrate testosterone levels (median ~10 ng/dL) without any need for Relugolix dose adjustment [15].

Subgroup Analysis of Demographics From the HERO III Trial

Multiple subgroup analyses from the pivotal HERO Phase III trial have evaluated the efficacy and safety of Relugolix versus Leuprolide across diverse patient populations. A geographical analysis by Bossi et al. demonstrated that Relugolix achieved rapid and sustained testosterone suppression across all regions studied. These included North America, Europe, and Asia. Castration rates consistently above 95% by day 29 and maintained through week 48 were demonstrated. This was comparable to results of the global HERO population (difference in sustained castration rate = 7.9%; 95% CI = 4.1-11.8; p < 0.0001) [16].

In the age-based subgroup analysis by Cookson et al., Relugolix maintained efficacy across all age groups. For men aged <65 years, the difference in sustained castration rates between Relugolix and leuprolide was 6.8% (95% CI = -4.3-17.9), and for those aged ≥65 years, 8.2% (95% CI = 4.2-12.2). Similar trends were seen when stratified at age 75 years, with differences of 6.3% (95% CI = 1.7-10.8) for ≤75 years and 12.1% (95% CI = 5.0-19.2). These results suggest that advancing age did not reduce treatment efficacy [17].

The analysis by George et al. examined the impact of Relugolix in Afro-Caribbean men. Results showed sustained castration rates of 93.3% (95% CI = 75.9-98.3) for Relugolix and 93.3% (95% CI = 61.3-99.0) for Leuprolide (95% CI = -15.5-15.5). Cardiovascular outcomes were consistent with the overall HERO findings, suggesting a lower incidence of MACE with Relugolix [18].

When outcomes were evaluated according to baseline body mass index (BMI) by Saad et al., Relugolix achieved rapid testosterone suppression across all BMI categories (<25, 25-29.9, ≥30 kg/m²). Efficacy and safety were comparable to the overall population, although specific CIs and p-values were not reported [19].

Finally, among Asian men, Ye et al. found that Relugolix achieved and maintained castration levels comparable to Leuprolide across the treatment period. No new safety concerns or clinically significant regional differences were demonstrated [20].

Patient-Reported Quality of Life Outcomes

Tombal et al. compared Relugolix with Leuprolide in men with advanced prostate cancer, looking at data from the HERO III trial. The study utilized a variety of patient-reported outcomes to assess patient health-related quality of life (HRQOL). Using the European Organisation for Research and Treatment of Cancer Quality of Life Core Questionnaire (EORTC QLQ-C30) and European Organization for Research and Treatment of Cancer Quality of Life Questionnaire-Prostate Module 25 tools, the study found no statistically significant differences in HRQOL after 48 weeks.

During the testosterone-recovery phase, patients on Relugolix reported lower hormone-related symptom scores. Supporting data showed an HR of 0.89 (95% CI = 0.65-1.22; p = 0.465), confirming no significant difference during treatment. Overall, Relugolix provided similar HRQOL to Leuprolide [21].

Pooled Safety Analysis

Bailen et al. performed a pooled safety analysis of two RCTs. The two trials included in the analysis were the HERO trial and the C27002 randomized phase 2 study. Overall, adverse event rates were similar between groups (92.9% with Relugolix vs. 93.5% with Leuprolide), with significant events occurring in 18.0% and 20.5% of patients, respectively. Common adverse events included hot flushes, fatigue, constipation, diarrhea, and arthralgia. The incidence of MACE was significantly lower with Relugolix (2.9%) compared to leuprolide (6.2%), with an HR of 0.46 (95% CI = 0.24-0.88) [22].

The REVELUTION Trial

The REVELUTION trial by Patel et al. compared Relugolix and Leuprolide in combination with radiotherapy for localized prostate cancer. Quality of life assessments using the International Prostate Symptom Score (IPSS) and the Expanded Prostate Index Composite (EPIC-CP) revealed that the Relugolix group experienced less acute urinary toxicity with a mean difference in IPSS urinary score of −3.68 (95% CI not reported; p = 0.04). However, there were no statistically significant differences between the two groups in the EPIC-CP domains of urinary incontinence, bowel function, sexual function, or vitality (all p > 0.4) [23].

Discussion

The collective evidence from recent clinical trials, subgroup analyses, and pharmacokinetic modelling strongly supports the efficacy of oral Relugolix. Data demonstrates that Relugolix is a viable alternative to traditional injectable ADTs in the management of advanced prostate cancer.

The pivotal HERO trial by Shore et al. demonstrated that Relugolix achieved castration-level testosterone (<50 ng/dL) in 96.7% of patients by day 15. This significantly outperformed Leuprolide (88.3%) in speed of suppression [6]. These findings are clinically significant as they exhibit how rapid testosterone suppression can be achieved with oral therapy. This may be beneficial in clinical scenarios requiring urgent hormonal control. The trial used randomization and had a large sample size, which adds to the strength of the evidence. However, the 2:1 randomization provides a greater data set for Relugolix when compared to Leuprolide. However, 48 weeks is also a relatively short duration and limits the assessment of long-term outcomes and safety.

Saad et al. expanded on these findings by evaluating long-term outcomes, including sustained castration rates and CRFS over a 48-week period. While the sustained castration rate numerically favored Leuprolide (97.0% vs. 91.7%), the confidence interval (−13.7%, 3.0%) indicated no statistically significant difference. Similarly, CRFS rates were comparable between groups, with HRs close to unity (HR = 1.03; 95% CI = 0.72-1.49; p = 0.89), suggesting equivalent long-term disease control [7]. While this study provides more data on the long-term efficacy of Relugolix, the CIs slightly favor Leuprolide.

Subgroup analyses by George et al. further reinforced evidence of Relugolix’s clinical efficacy. Patients receiving concomitant therapies such as docetaxel and enzalutamide maintained castration-level testosterone suppression in combination with Relugolix. These findings indicate that Relugolix can be effectively integrated into multimodal treatment regimens. Importantly, cardiovascular safety was notably improved with Relugolix [8]. MACE occurred in only 2.9% of patients compared to 6.2% in the Leuprolide group [6]. This finding is particularly important given the high morbidity of cardiovascular complications in patients with prostate cancer. The use of a subgroup design limits the ability to determine causal inference. However, the improved cardiovascular safety demonstrated by Relugolix is clinically relevant and requires dedicated research. This study only considered docetaxel and enzalutamide in combination with Relugolix and Leuprolide. As such, it does not provide any data for abiraterone, another common agent used in the management of advanced prostate cancer.

Spratt et al. explored the role of Relugolix in combination with radiotherapy, a common approach in locally advanced and high-risk disease. Their analysis revealed no significant difference in CRFS between Relugolix and Leuprolide (HR = 0.97; 95% CI = 0.35-2.72; p = 0.62), supporting the use of Relugolix in this setting. Stratification by ADT duration showed high castration rates in both short-term (95%) and long-term (97%) Relugolix cohorts, further validating its consistency across treatment timelines [9]. As a post hoc analysis, this study provided data on combination therapy in the treatment of prostate cancer. The power of the study was limited by wide CIs and non-significant HRs.

In addition to the above, Gallagher et al. demonstrated significant rates of castration and PSA level declines in patients receiving neoadjuvant/adjuvant Relugolix with SBRT [12]. The results, however, are limited by their small sample size (n = 52), which reduces statistical power. There was also no control group and no randomization. This does not allow for any direct comparison between ADT regimes. The short follow-up focused only on early biochemical markers without assessing long-term morbidity/mortality outcomes. Additionally, limited adverse event reporting and potential lack of population diversity further reduce the applicability of the findings to broader clinical practice.

The RCT Dearnaley et al. included 103 patients undergoing radiotherapy in combination with ADT. Relugolix demonstrated faster castration and better testosterone recovery than Degarelix with comparable adverse events. Notably, the median time to testosterone suppression was markedly shorter with Relugolix compared with Leuprolide (4 days vs. 29 days) [11]. The study had a relatively small sample size and lacked any blinding, which may have introduced bias. However, it provides valuable head-to-head data for oral versus injectable GnRH antagonists.

Further supporting the above clinical findings, a pharmacokinetic/pharmacodynamic model developed by Lee et al. provided pharmacological data. Simulations confirmed that Relugolix achieves castration-level testosterone by day two and profound castration (<20 ng/dL) by day nine. Additionally, testosterone suppression was maintained in 97.3% of patients even after a seven-day treatment interruption [10]. While modelling studies provide information regarding pharmacodynamic/pharmacokinetic data, it is simulated data. As such, they may not be fully reflective of real-world clinic outcomes.

In addition to its efficacy, Relugolix has demonstrated a favorable safety profile across multiple trials. The most common adverse event was hot flushes (50-60%), followed by mild gastrointestinal symptoms such as diarrhea, which were more frequent than with Leuprolide but generally non-disruptive [14]. Fatal adverse events were less frequent with Relugolix (1.1% vs. 2.9%) [13]. Severe adverse events were infrequent (<5%) and included isolated cases of hypertension and headache [11]. These findings show Relugolix’s potential as a well-tolerated oral ADT option, particularly for patients with cardiovascular comorbidities or those undergoing combination therapy. A pooled safety analysis by Bailen et al. found minimal difference in adverse events across two RCTs in patients receiving Relugolix versus Leuprolide. The results, however, did demonstrate a lower rate of cardiovascular complications [22].

Brown et al. concluded that standard-dose Relugolix effectively maintains castration when co-administered with apalutamide and that the combination has a favorable short-term safety profile. However, the small sample size, lack of a comparator arm, and short follow-up limit conclusions about long-term clinical outcomes [15].

Multiple subgroup analyses of patients from the HERO III trial confirm the robust and consistent efficacy of Relugolix in achieving sustained testosterone suppression across age, race, geography, and body composition, while maintaining a favorable safety profile consistent with the global HERO population [17-20]. While these subgroup analyses were informative, they are limited by relatively small sample sizes within some subgroups. This reduces the statistical power to detect significant differences.

Finally, two studies evaluating patient-reported quality of life outcomes showed favorable results for Relugolix compared to Leuprolide. The HERO III trial showed no significant differences in overall quality of life between Relugolix and Leuprolide [21]. Meanwhile, the REVELUTION trial found Relugolix significantly reduced acute urinary toxicity during radiotherapy for localized disease (mean IPSS difference = −3.68, p = 0.04). Other quality of life domains were similar, suggesting Relugolix offers comparable outcomes with a modest urinary benefit [23].

Limitations

This review is limited by the relatively small number of primary research articles available for inclusion. Of the 84 studies initially identified, only 18 met the inclusion criteria. As such, pooled statistical analysis was limited, and results should be interpreted cautiously. Additionally, while cardiovascular safety outcomes were discussed, this was not the primary focus of the review. A comprehensive analysis of adverse events was beyond the scope of this work. Further limitations include potential publication bias, as only English-language articles from PubMed-indexed journals were considered. Heterogeneity across studies in terms of design, endpoints, patient populations, and duration of follow-up also limits the ability to draw definitive conclusions. Long-term efficacy and safety data on Relugolix, particularly in diverse real-world populations and in combination with other treatment modalities, remain limited. A meta-analysis was not performed due to the heterogeneity of the research articles included, and only a subset of the final 18 papers included comparator arms suitable for meta-analysis.

## Conclusions

Relugolix is a clinically effective oral GnRH antagonist for the treatment of advanced prostate cancer. It provides rapid and sustained suppression of testosterone levels, as demonstrated by evidence from recent clinical trials and pharmacokinetic modelling studies. Its efficacy is comparable to traditional injectable ADTs such as Leuprolide and Degarelix. Relugolix offers the advantage to patients of being an oral formulation over an injectable one. It has also been demonstrated to have a more favorable cardiovascular safety profile. Studies have demonstrated its effectiveness as a neoadjuvant/adjuvant therapy alongside radiotherapy and systemic agents such as docetaxel and enzalutamide, further supporting its role management of prostate cancer. However, long-term outcomes, including survival benefits and quality of life impacts, require further investigation. While current data are promising, ongoing studies are needed to fully establish the role of Relugolix long-term in the management of prostate cancer.
